# Overexpression of HE4/WFDC2 gene in mice leads to keratitis and corneal opacity

**DOI:** 10.1515/biol-2025-1234

**Published:** 2025-12-29

**Authors:** Lingjin Tuo, Taojun Zhang, Xiaolun Xu, Jian Lin, Fengchao Wang, Hao Xu, Shi-Wen Jiang

**Affiliations:** Lianyungang Research Institute for Women’s and Children’s Health, Lianyungang Maternal and Child Health Hospital Affiliated to Kangda College of Nanjing Medical University, Lianyungang, Jiangsu Province, China; Department of Interventional Imaging, The First Affiliated Hospital of Shanxi Medical University, Taiyuan, Shanxi Province, China; Department of Ophthalmology, Lianyungang Maternal and Child Health Hospital Affiliated to Kangda College of Nanjing Medical University, Lianyungang, Jiangsu Province, China; National Institute of Biological Sciences, Beijing, China; Department of Gynecology, Lianyungang Maternal and Child Health Hospital Affiliated to Kangda College of Nanjing Medical University, Lianyungang, Jiangsu Province, China

**Keywords:** HE4 overexpression, keratitis, corneal opacity, WFDC2, keratopathy

## Abstract

HE4 is overexpressed in malignant lesions, and elevated serum HE4 levels have been applied as a biomarker for gynecologic cancers. While previous studies have demonstrated the HE4 activities in cancer biology, its role(s) in benign disease is unclear. In current study, we characterize the keratopathy phenotype of transgenic mice with HE4 overexpression (HE4-OE). HE4-OE mice started to display signs of keratitis such as eye-scratching, conjunctiva inflammation, red eyes, periocular secretions, and rough skin/hair loss around the eyes at 3 months after birth. All the mice suffered keratitis, severe corneal opacity and ELISA results indicated HE4 overexpression, and significantly increased IL-6 and TNF-α levels in the cornea. Immunostaining demonstrated the accumulation of disorganized collagen fibers, fibroblast activation, and the presence of vessel-like structures, indicating the progression of corneal opacification. The cornea alkali burn model showed increased HE4 expression, which was accompanied by the elevation of IL-6 and TNF-α in the cornea. Thus, both the HE4-OE and alkali burn models have correlated increased HE4 expression to inflammatory responses. These studies indicate that HE4 may play a significant role(s) in keratopathy and other physiopathological conditions of the eye.

## 1 Introduction

The cornea is a thin, transparent, elastic, and disk-like membrane located at the foremost part of the eyeball. Histologically, the cornea consists of five layers: corneal epithelium, Bowman’s Layer, stroma, lamina elastica posterior, and endothelium, from the front to back. By its direct exposure to environments, the cornea is susceptible to injury and microbial infection. Affecting more than four million people globally, corneal opacity represents a major cause of visual disorder and blindness [1]. Injury, surgery, infection, and aging are risk factors for corneal opacity [2], 3]. Corneal opacity is characterized by the deposition of collagen fibers, fibroblast hyperplasia, and angiogenic activity [4]. Although clinically corneal opacity cases are classified into the hereditary, infectious, malnutritional, and traumatic categories [5], pathologically many cases of corneal opacity are accompanied by the progressive keratitis [6]. Keratitis often manifests as pain, dryness and redness around the eyes. It is noteworthy that due to the frequent use/abuse of contact lenses, and the increasing cases of ophthalmological surgery/corneal transplantation/HIV infection/diabetes, the incidence rate of keratitis is on the rise. If not properly treated, the recurrent inflammatory attacks associated with keratitis can lead to progressive corneal opacity and irreversible loss of vision [7].

HE4 (human epididymis protein 4) is a secretory glycoprotein encoded by the WFDC2 gene. The prototype transcript of WFDC2 gene was first detected in the distal section of human epididymis [8]. Later studies showed that HE4 mRNA is highly expressed in the trachea and salivary gland, and low levels of HE4 mRNA are present in the lungs, prostate, pituitary gland, thyroid, kidney and other tissues [9]. Moreover, HE4 mRNA and protein were found to be overexpressed in many types of malignant lesions such as those of ovarian, endometrial, breast, lung, and esophageal cancers [9], [10], [11]. Serum HE4 is a cancer biomarker for the early detection, auxiliary diagnosis and prognosis of gynecologic cancers [12]. Moore et al. created the Risk of Ovarian Malignancy Algorithm (ROMA) based on the serum HE4 level [13] that can effectively predict epithelial ovarian cancer in women with pelvic masses. In addition, increased serum HE4 levels were found to be associated with nonmalignant diseases such as kidney fibrosis [14], multiple sclerosis [15], autoimmune disorders, and heart failure [16]. Elevated circulatory HE4 levels were also observed under conditions of inflammation, aging, smoking, and obesity [17], [18], [19]. Thus, HE4 overexpression represents a physiopathological event and the creation/characterization of *in vivo* HE4 overexpression models through gene manipulation are of particular biological significance.

HE4 protein contains the whey four-disulfide core (WFDC) domain and possesses a cross-class protease inhibitory activity capable of binding and inhibiting the endogenous serine, aspartyl and cysteine proteases. Overexpression of HE4 in several cancer cell lines promoted cell proliferation, cell migration and invasion, and suppressed cell apoptosis [20]. Moreover, overexpression of HE4 in endometrial as well as ovarian cancer cell lines promoted tumor growth [21], 22] and metastasis [23] in mouse xenograft models. In contrast, knockdown of HE4 expression inhibited cell proliferation and migration, increased cell apoptosis [24], and enhanced the cancer cells’ sensitivity to therapeutic drugs [25], 26]. Interestingly, recent studies suggested that HE4 may serve as a pro-inflammatory mediator [27] which can be used as a novel biomarker for inflammatory response [28]. Although previously HE4 has not been involved in keratopathy and/or vision disorders, WFDC1, another gene encoding the WAP-type four disulfide core domain-containing endogenous protease inhibitor, was reported to be constitutively expressed in the tissues of mouse eye, and its mutation was associated with multiple ocular defects (MOD) in cattle [29].

Previous studies have shown that knockout of HE4 gene in mice impaired lung development, leading to severe postnatal dyspnea and postnatal death 24 h after birth [30]. Mice engineered to overexpress HE4 (HE4-OE) exhibited structural anomalies of seminiferous tubules, Leydig cell hyperplasia and spermatogenesis impairment [31]. The current study focuses on the ophthalmological phenotype of the HE4-OE mice. Characterization of the HE4 role(s) in the development of keratitis and corneal opacity will help us to better understand the pathogenesis of these diseases.

## 2 Materials and methods

### 2.1 Materials

NaOH was purchased from Sangon Biotech (Cat^#^ C601009-0500, Shanghai, China). The Animal anesthesia machine was a product of RWD Life Science (Cat^#^ R500IP, Shenzhen, China). Isoflurane was purchased from RWD Life Science (Cat^#^ R510-22-10, Shenzhen, China). Polyclonal rabbit antibodies against mouse HE4 (Cat^#^ ab273130), TNF-α (Cat^#^ ab183218), IL-6 (Cat^#^ ab233706), KI67 (Cat^#^ ab92742), Pax6 (Cat^#^ ab195045), Keratin12 (Cat^#^ ab185627), CD31 (Cat^#^ ab76533), *α*-SMA (Cat^#^ ab242395), FSP1 (Cat^#^ SAB5700127), and elastin (Cat^#^ AB2043) were purchased from Abcam (Cambridge, UK) and Merck KGaA (Darmstadt, Germany). The horseradish peroxidase-labeled goat anti-rabbit IgG was purchased from ZSGB-Bio (Cat^#^ ZB-2301, Beijing, China). The Alexa Fluor™ 488-labeled goat anti-rabbit IgG was a product of Thermo Fisher Scientific (Cat^#^ A11070, USA). Triton 8-100^®^ and Bovine Serum Albumin (BSA)^®^ were purchased from Sigma-Aldrich (Cat^#^ X100 and Cat^#^ V900933, St. Louis, MO). TBS powder (pH 7.4) was purchased from Sangon Biotech (Cat^#^ A510025, Shanghai, China). BCA Protein Quantification Kit was purchased from Beyotime (Cat^#^ P0012, Shanghai, China). Mouse HE4 ELISA Kit was purchased from Abcam (Cat^#^ ab245724, Cambridge, UK). Mouse TNF-α ELISA Kit and Mouse IL-6 ELISA Kit were purchased from Yaenzyme (Cat^#^ VAL609 and Cat^#^ VAL604, Shanghai, China). PrimeScript™ FAST RT Reagent Kit with gDNA Eraser was purchased from Takara (Cat^#^ RR092A, Kyoto, Japan).

### 2.2 Animal

The homozygous HE4 overexpression mouse model (HE4-OE) was created on the C57BL/6 background with the CRISPR/Cas9 technique as previously reported [31]. The 8-week-old specific pathogen-free (SPF) grade C57BL/6J mice were provided by GemPharmatech (Jiangsu, China). Mice were kept in an SPF facility, maintained under 22 °C, 70 % humidity, and automatic light control (12 h light/12 h dark). Mice were fed *ad libitum* with sterilized feed. Six-month-old female and male HE4-OE mice that have developed keratopathy and wild-type mice were sacrificed, and the corneal tissues were collected for histological studies.

To create the alkali burn model, a circular filter paper (11 μm pore size) with a diameter of 2 mm was immersed in 1.0 M of NaOH. Twelve-week-old female and male C57 mice were anesthetized with isoflurane. The filter paper soaked with NaOH was placed on the mouse corneas for 30 s. Mice were sacrificed at 3, 9, and 24 h after alkali burn (sample size *N* = 3 for each time point), the cornea tissues were destructed for mRNA and protein isolation. For histological studies, the eyeballs were isolated at 12 h after alkali burn (sample size *N* = 3).

**Ethical approval:** The research related to animal use has been complied with all the relevant national regulations and institutional policies for the care and use of animals, and has been approved by the Animal Ethics Committee of Kangda College of Nanjing Medical University (License number: IACUC-24XS001).

### 2.3 Hematoxylin-eosin staining (H&E), immunohistochemistry (IHC), and immunofluorescence staining

Mouse corneal tissues were collected and fixed with 4 % paraformaldehyde solution for 24 h. The corneal tissues were dehydrated with 70 %, 90 % and 100 % ethanol sequentially, 1 h for each step. Corneal tissues were cleared by immersing in the xylene-ethanol mixture (1:1, v:v) for 2 h and in 100 % xylene twice, each for 2 h. Tissues were transferred to melted paraffin to form tissue blocks. Tissue sections with a thickness of 4 μm were cut and mounted onto glass slides. Tissue sections were deparaffinized with 100 % xylene for three times and rehydrated sequentially with 100 %, 95 %, 80 %, 70 % and 50 % ethanol, each for 5 min. After rehydration in PBS for 5 min, tissue sections were stained with hematoxylin for 20 min. After destaining with HCl-ethanol solution (1 % HCl in 70 % ethanol) and rinsing with distilled water, tissue sections were stained with eosin for 3 min. Following dehydration with 70 %, 80 % and 90 % ethanol sequentially, stained tissue sections were cleared with xylene.

For IHC, the tissue sections were deparaffinized and rehydrated as described above. The sections were boiled in 0.01 M citrate solution for 6–8 min for antigen retrieval. After cooling to room temperature, the tissues were treated with 3 % H_2_O_2_ for 10 min to quench the endogenous peroxidase. The sections were covered with 5 % BSA and incubated at 37 °C for 30 min to block nonspecific reactions. The rehydrated cornea sections were stained with rabbit anti-mouse HE4 antibody (1:350) for 1 h at room temperature. After washing with TBS for three times, goat anti-rabbit secondary antibody was applied at 1:400 dilutions for 1 h. Color development was performed with DAB Substrate (IHC-101, Bethyl Laboratories, Inc., Montgomery, TX, USA). Counterstaining was carried out with Gill’s Hematoxylin Solution (Cat. No. SC-24973, Santa Cruz Biotechnology, Dallas, TX, USA), following the manufacturer’s instructions. The stained sections were observed and photographed under a microscope (Nikon, Y-TV55, Japan) with 20× and 40× objective lens.

For immunofluorescence staining, the tissue sections were deparaffinized, rehydrated, boiled for antigen retrieval, and blocked with BSA, following the above-described procedures. Rabbit anti-mouse antibodies against the following protein markers (dilution), HE4 (1:350), TNF-α (1:5000), IL-6 (1:50), KI67 (1:250), Pax6 (1:350), Keratin12 (1:50), FSP1 (1:250), elastin (1:80), CD31 (1:500) and *α*-SMA (1:150), were applied respectively. Incubation with primary antibodies was carried out overnight at 4 °C. The sections were rinsed with Tris Buffered Saline (TBS) and Tris Buffered Saline with Tween 20 (TBST) for four times each. The Alexa Fluor™ 488-labeled goat anti-rabbit IgG secondary antibody was added and incubation continued for 1 h at 37 °C. Following rinsing with TBS for four times, tissue sections were counterstained with DAPI (0.1 μg/ml for 10 min). Corneal sections were observed under an inverted fluorescence microscope at 20× and 40× objective magnifications.

### 2.4 Masson staining

Each deparaffinized and rehydrated corneal section was stained with 50 μl hematoxylin solution for 5 min. After rinsing with distilled water, tissue sections were stained with 50 μl of Ponceau S staining solution for 10 min. Following treatment with the phosphomolybdic acid solution for 2 min, the tissue sections were dehydrated in 70 %, 80 %, 90 % and 100 % ethanol sequentially, each for 10 Sec. Tissue sections were immersed in 100 % xylene solution twice for 2 min each time. Stained sections were examined and photographed at 20× and 40× objective magnifications (Nikon, Y-TV55, Japan).

### 2.5 ELISA

Each mouse corneal tissue was minced with a pair of scissors and homogenized with the Dounce tissue grinder in 100 µl of PBS solution. After two cycles of freezing at −80 °C and thawing at 4 °C, the mixture was centrifuged at 12,000*g* for 5 min for 4 °C, and supernatants were collected. Protein concentrations were determined with the BCA method. The Mouse HE4 ELISA Kit was used to measure HE4 protein levels in the cornea tissue extracts. Eight μg of protein extracts were used to measure HE4 protein levels, and preliminary experiments with the kit showed that the HE4 readouts at this amount of input fell in the linear range. Eight μg of protein in 10 μl were added to each well of the 96-well plate. After incubation at room temperature for 2 h, protein solution was aspirated from wells, and wells were washed 4 times with the 0.4 ml of washing buffer. Antibody Cocktail (0.1 ml) was added to each well and removed after incubation at room temperature for 1 h. Following three times of washing with washing buffer, 50 μl of substrate solution were added to each well. After incubation in dark at room temperature for 30 min, 100 μl of stop solution were added, and absorbance at 450 nm was determined within 30 min on the Microplate Reader (Thermo Scientific, Cat^#^ A51119700DPC, USA). TNF-α and IL-6 were measured with similar procedures. All the ELISA results were converted to the amount of target protein per amount of total protein input (pg/mg) with the use of standard curves.

### 2.6 RT-qPCR

Mouse corneal tissues were minced with a pair of scissors and homogenized with the Dounce tissue grinder in 100 µl of distilled water. Total RNA was isolated with the RNA Rapid Extraction Kit (Takara, Kyoto, Japan) following the manufacturer’s instructions. RNA concentrations were determined on a Nanodrop photometer (Thermo Scientific, USA). Reverse transcription was performed with the use of PrimeScript™ FAST RT Reagent Kit with gDNA Eraser. Real-time PCR was carried out on the Applied Biosystem 7500 (ThermoFisher Scientific, USA) in a total volume of 25 μl that contained 2.5 μl of reverse transcription products, 1 μl of forward and backward primers each, and 12.5 μl of reaction mix. The designations and sequences of PCR primers are documented in Supplementary Table 1. PCR conditions are: initial denature at 95 °C for 5 min, followed by 40 cycles of denature at 95 °C for 5 s and amplification at 60 °C for 10 s. The PCR products were resolved in agarose gel electrophoresis to verify specific amplification of target cDNA. The threshold cycle number of the target gene was standardized by that of *GAPDH* gene as an internal reference. Final results for each target mRNA were expressed as the relative ratios between HE4-OE and control group, which set the control group to 1.

### 2.7 Statistical analysis

All quantitative data were expressed as means ± STDEV. Statistical analyses were performed using SPSS Statistics 25.0. Two-tailed *t*-test was performed to evaluate the differences between the experimental and control groups. *p* < 0.05 was used as the criterion for statistical significance.

## 3 Results

### 3.1 The keratopathy of HE4-OE mice

**Figure 1:**
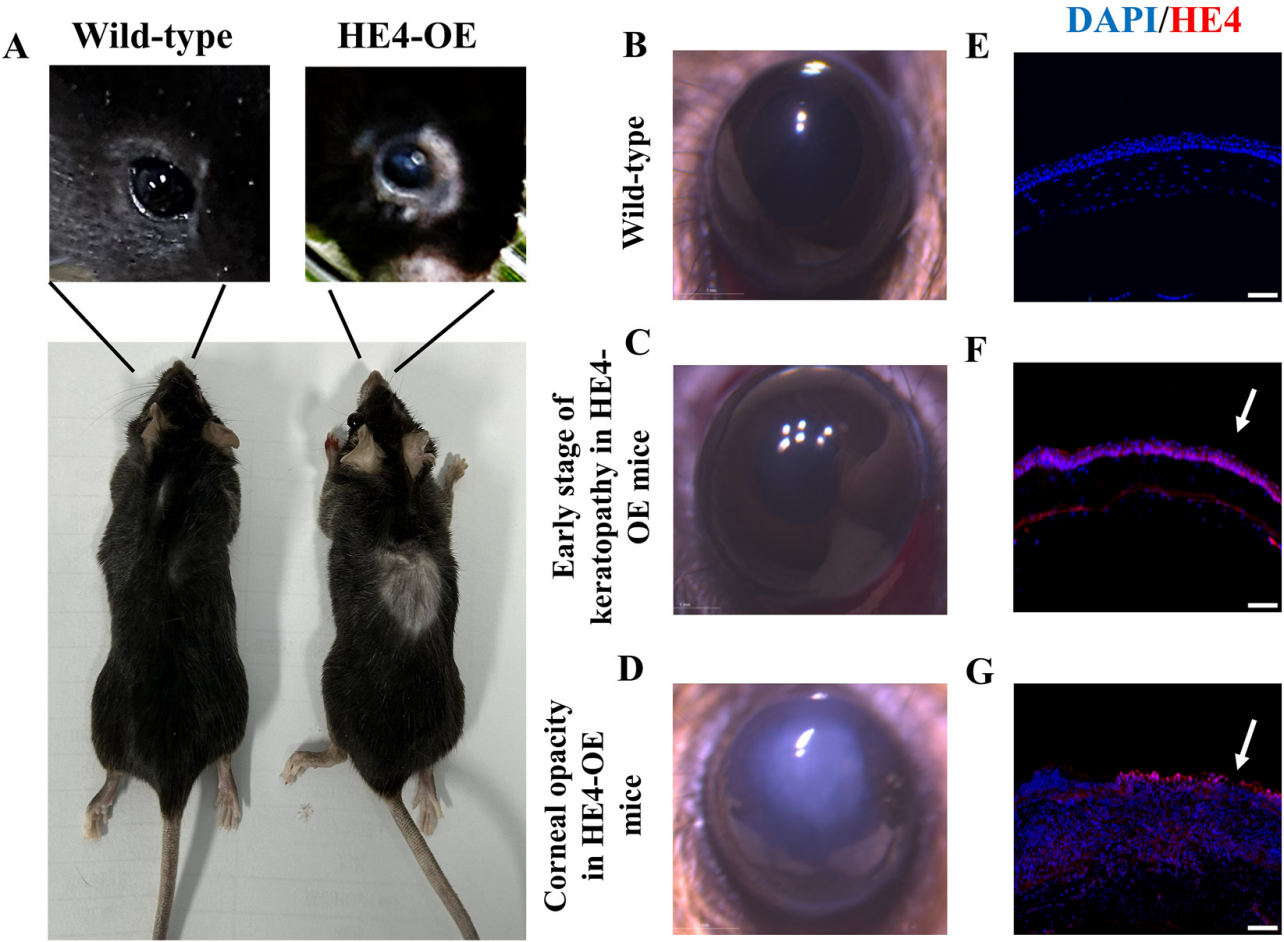
The keratopathy of HE4-OE mice. A. Corneal opacity was observed in HE4-OE mice. The left is a representative photograph of a 6-month-old wild-type mouse, and the right is a representative photograph of a 6-month-old HE4-OE mouse. Note the opaque and untransparent look of the cornea, and hair loss around the eye and at the back, of the HE4-OE mouse. B. C. and D. are stereomicroscopic photographs of the corneas of a wild-type mouse, a 3-month-old HE4-OE mouse before development of opacity, and a 6-month-old HE4-OE mouse with the development of opacity, respectively. Correspondingly, E. F. and G. are microscopic pictures of HE4 antibody-stained corneal sections for the wild-type mouse, the HE4-OE mouse before development of opacity, and the HE4-OE mouse with the development of opacity, respectively. Note the diminished HE4 expression in the cornea of wild-type mice, but high HE4 expression in the corneal epithelium before development of opacity (indicated by arrow). The development of opacity in HE4-OE was accompanied by an increased HE4 expression in the corneal epithelium and stroma (indicated by arrow). Scale bars = 50 µm, *N* = 3, two female mice and one male mouse.

As previously reported, the homozygous HE4-OE males had a modestly increased serum HE4 level (2.7-fold) and exhibited spermatogenesis impairment [31]. Importantly, these mice started to exhibit the signs of keratitis, such as eye-scratching, conjunctiva redness, red eye, massive periocular secretions, and rough skin/inflammation/hair loss around the eyes (Figure 1A) at 3 months after birth. While wild-type and heterozygous HE4-overexpression mice had no phenotypic alteration, about 50 % of HE4-OE mice, with no gender difference, developed keratitis in at least one eye at 3 months after birth, and at one year of age, they all had keratitis. Both eyes were equally affected with no difference between the left and right eyes. While the corneas of wild-type mice were transparent (Figure 1B) and the corneas of HE4-OE mice also remained transparent initially (Figure 1C), at 2–3 months following the onset of keratitis the cornea of affected eye turned opaque as shown under the stereomicroscope (Figure 1D). As the disease progressed, HE4-OE mice first developed opacity in the central area of the cornea, and the opaque area gradually expanded to the entire cornea. Eventually, all HE4-OE mice developed severe corneal opacity. The mice suffering severe corneal opacity relied on olfactory and tactile senses to locate food and water, and had no blink response when an object approached the eyeball closely. Thus, the keratopathy appeared to lead to a significant loss of vision.

Immunohistochemistry with HE4 antibody showed that while the corneas of wild-type mice had diminished HE4 expression (Figure 1E), the corneal epithelium of HE4-OE mice expressed high level of HE4 protein even before the occurrence of opacity (Figure 1F). When the keratopathy became apparent, HE4 expression in the corneal epithelium increased to a higher level (Figure 1G).

It is noteworthy that repeated inbreeding often nonspecifically causes genetic diseases, and such “spontaneous” ophthalmological disorders are not rare in some strains of transgenic mice [32]. In this case, however, the HE4-OE mice uniformly developed a specific keratopathy that followed a similar temporal pattern and no such phenotype was present in the wild-type littermate or C57 mice maintained under the same conditions. By these observations, the manifestations of keratitis and corneal opacity appeared to represent a specific phenotype of HE4-OE mice as a result of HE4 gene overexpression.

### 3.2 Histological analyses of the keratopathy in HE4-OE mice

**Figure 2:**
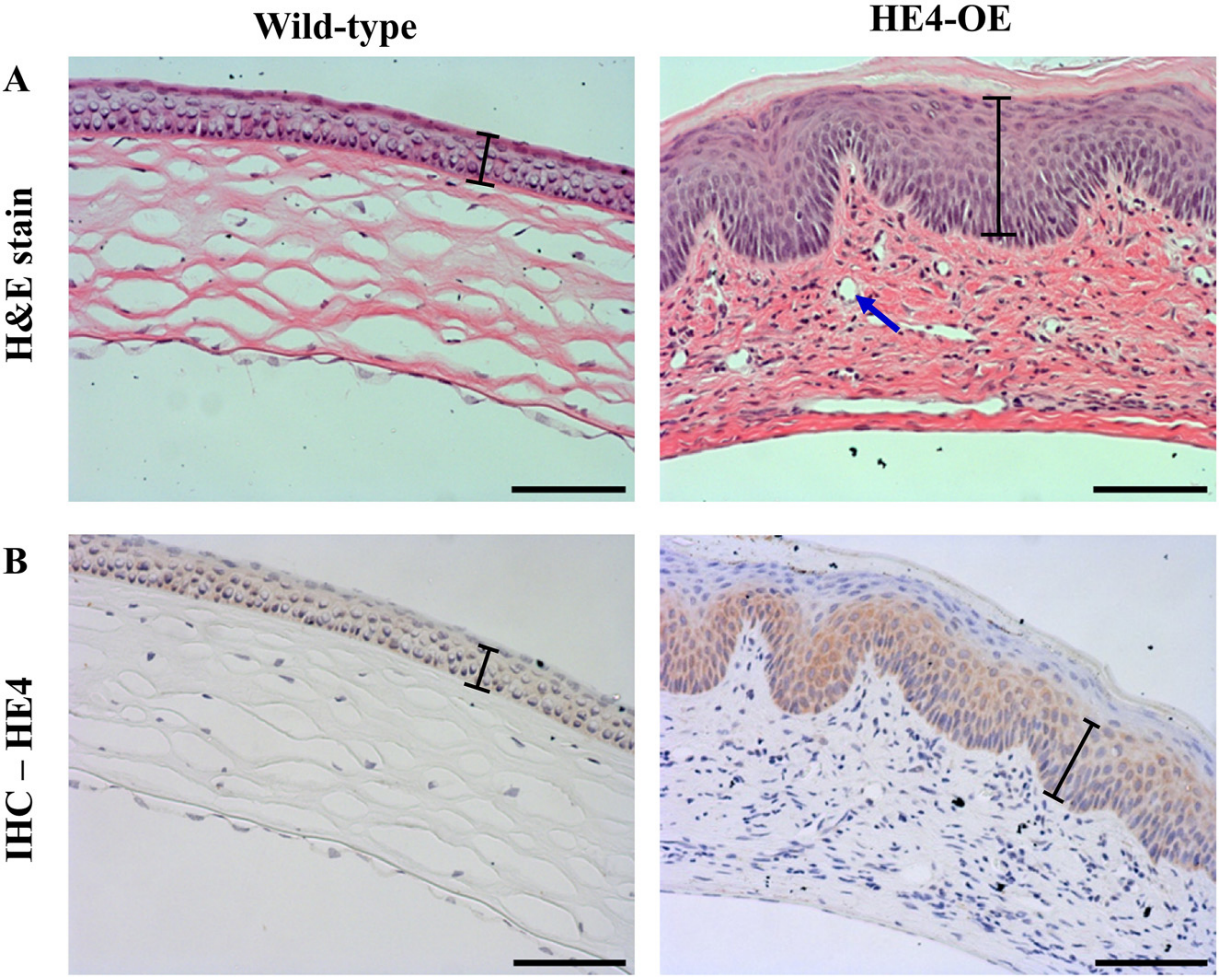
The keratopathy of HE4-OE mice. A. The histological structures of the corneas of 6-month-old wild-type and HE4-OE mice were examined after H&E staining. Note the increased thickness of the corneal epithelium (indicated by the black bars), increased cellular components, and the presence of multiple vessel-like structures (indicated by the blue arrow) in the cornea of a HE4-OE mouse. Scale bars = 50 µm, *N* = 3, two female mice and one male mouse. B. Immunohistochemistry with anti-HE4 antibody. Increased HE4 expression was observed in the corneal epithelium of HE4-OE mouse. Scale bars = 50 µm, *N* = 3, two female mice and one male mouse.

The results of H&E staining showed that compared to wild-type mice, the tear film layer of the cornea was much thicker in the HE4-OE mice with corneal opacity (Figure 2A). The corneal epithelium of HE4-OE mice also became thicker and uneven in thickness compared to that of wild-type mice. Moreover, the corneal stromal layer contained more cellular components as indicated by the presence of numerous dark-stained nuclei. While the collagen fibers in the corneas of wild-type mice were well-organized into a regular, net-like structure, they became disorganized and thick bundles heavily stained by eosin in the opaque corneas of HE4-OE mice. While the absence of blood vessels was verified in the corneas of wild-type mice, numerous vessel-like structures were observed in the stromal layer of the opaque corneas of HE4-OE mice (Figure 2 A). Immunohistochemistry with HE4 antibody confirmed that HE4 expression was significantly increased in the corneal epithelium of HE4-OE mice with keratopathy in comparison with the wild-type mice (Figure 2B).

### 3.3 Keratitis occurred in HE4-OE mice

**Figure 3:**
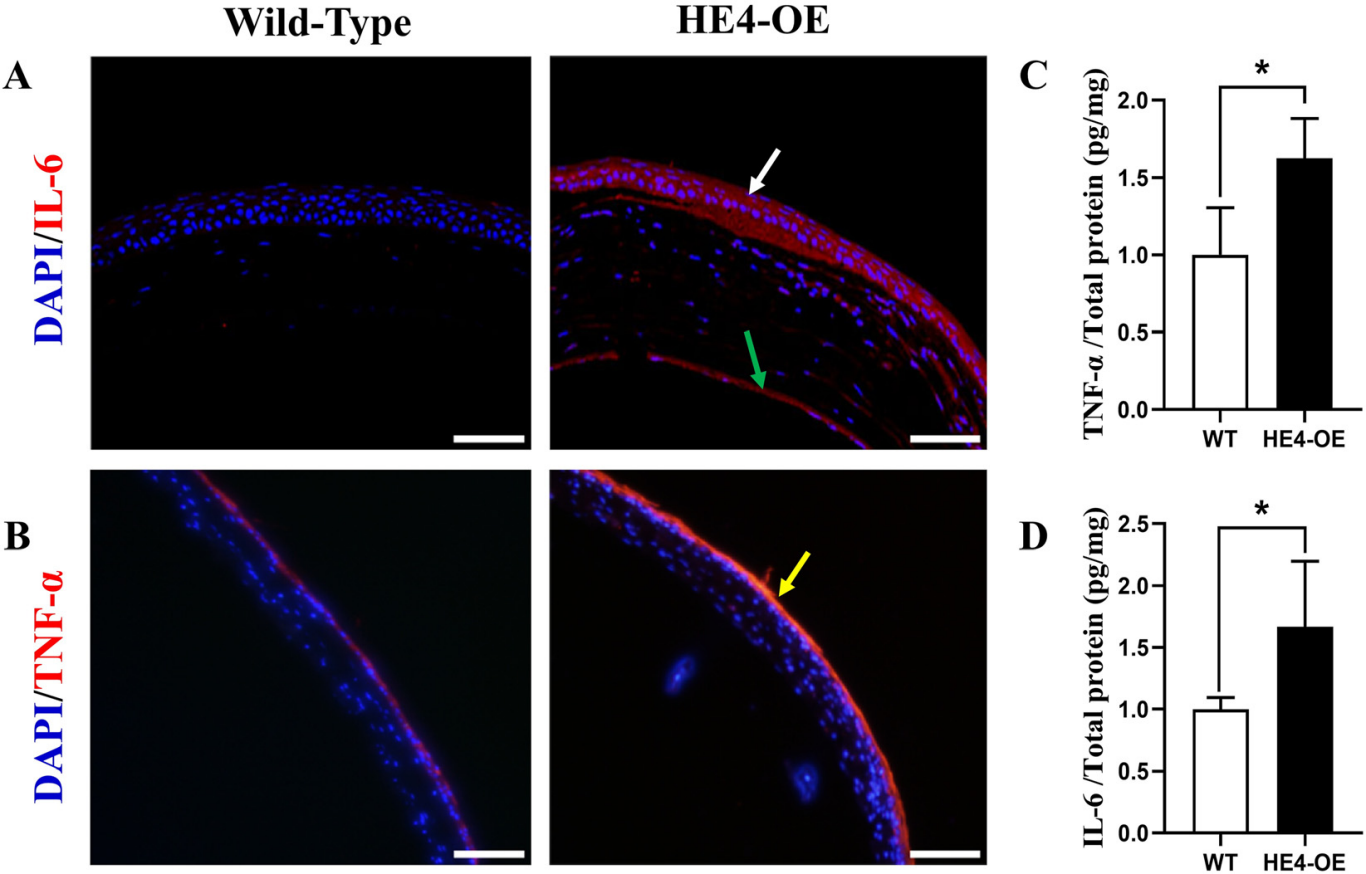
Keratitis of HE4-OE mice. A. and B.: The expression levels of pro-inflammatory factors IL-6 and TNF-α were detected in mouse corneas with specific antibodies. The white and green arrows point to increased IL-6 in the corneal epithelium and stromal fibroblasts of 6-month-old HE4-OE mice, respectively. The yellow arrow indicates increased TNF-α expression in the corneal epithelium of the HE4-OE mouse. Scale bars = 50 µm, *N* = 3, two male mice and one female mouse. C. and D.: IL-6 and TNF-α levels in the corneal protein extracts were determined with ELISA. Significantly increased IL-6 and TNF-α levels were found in the corneas of 6-month-old HE4-OE mice. ELISA results were individually standardized by the protein input of each sample, and compared between the two groups, *N* = 4, two male mice and two female mice, with the values of the wild-type set at 1. The data were expressed as mean ± SD. *p < 0.05.

To determine corneal inflammation in HE4-OE mice, immunohistochemistry was performed with antibodies against inflammatory markers IL-6 and TNF-α. No IL-6 expression was detected in the corneas of wild-type mice, but high levels of IL-6 expression in the corneal epithelium and low levels of IL-6 expression in the stromal layer were found in the corneas from HE4-OE mice (Figure 3A). Although a low level of TNF-α was present in the normal corneal epithelium of wild-type mice, a much increased TNF-α level was observed in the corneal epithelium of HE4-OE mice (Figure 3B). Also, quantitative measurement with ELISA indicated a significant elevation of IL-6 and TNF-α in the corneal protein extracts from HE4-OE mice in comparison with wild-type mice (Figure 3C and D). The specific numerical values and *p*-values were listed in the supplementary information. These observations indicated that the development of corneal opacity was accompanied by keratitis in HE4-OE mice.

### 3.4 Increased proliferation of epithelial and stromal cells in the corneas of HE4-OE mice

**Figure 4:**
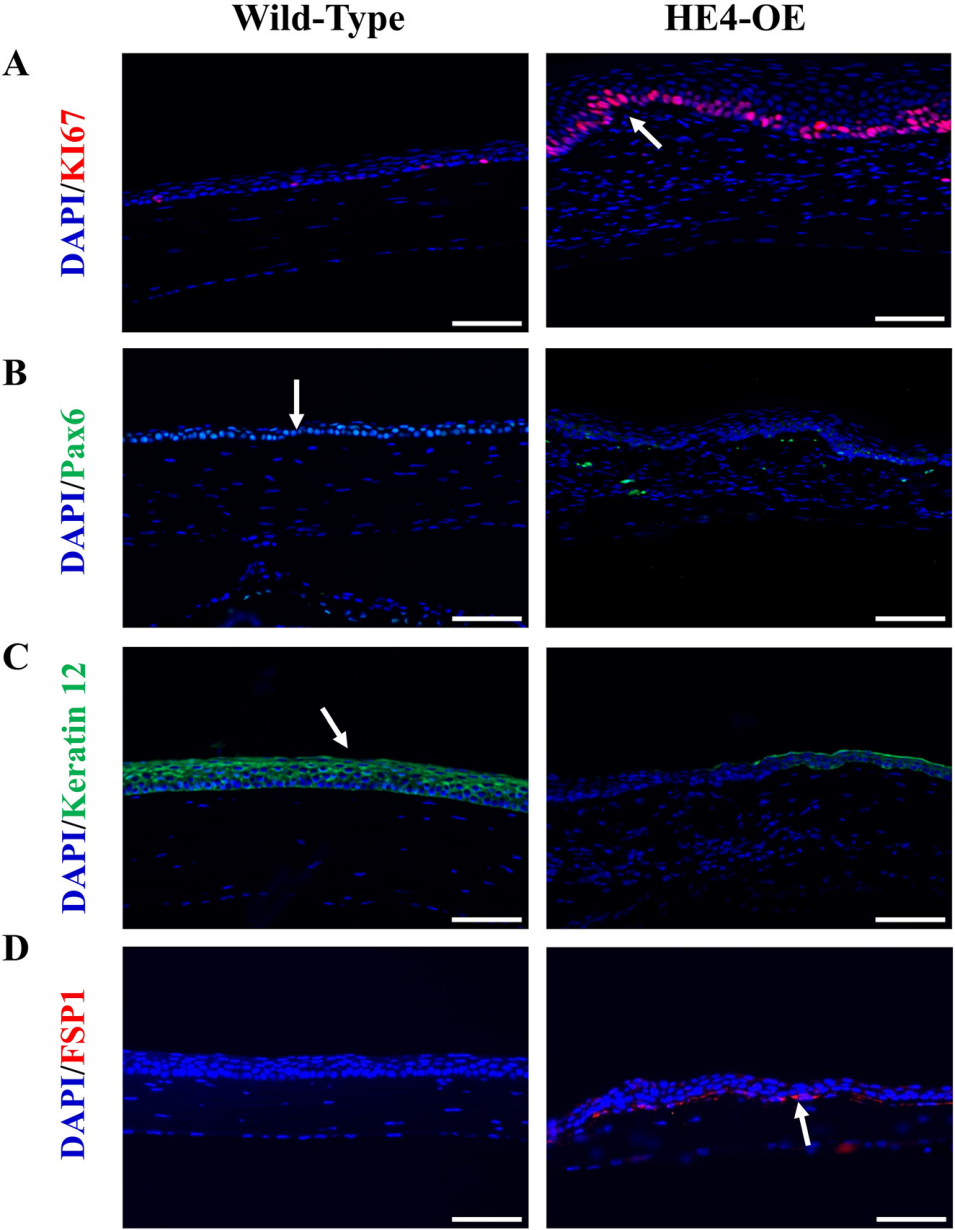
Increased proliferation, and dedifferentiation of epithelial cells, and fibroblast activation in the corneas of HE4-OE mice. A. Proliferation of corneal epithelial cells were determined by immunohistochemistry using specific antibodies against KI67, and cell nuclei were visualized by DAPI staining. An increased number of KI67-positive epithelial cells (stained in red fluorescence, indicated by white arrow) were detected in the corneas of 6-month-old HE4-OE mice. Scale bars = 50 µm, *N* = 3, two male mice and one female mouse. B. The epithelial cells in the cornea of wild-type mice were Pax6-positive (green fluorescence, indicated by white arrow). But the epithelial cells in the cornea of 6-month-old HE4-OE mice were Pax6-negative. Scale bars = 50 µm, *N* = 3, two male mice and one female mouse. C. The Keratin12-positive (red fluorescence, indicated by the white arrow) and Keratin12-negative epithelial cells in the cornea of 6-month-old wild-type and HE4-OE mice, respectively. Scale bars = 50 µm, *N* = 3, two male mice and one female mouse. D. In comparison with 6-month-old wild-type mice, multiple FSP1-positive fibroblasts (red fluorescence, indicated by the white arrow) were detected near the base of epithelial layer of HE4-OE mice. Scale bars = 50 µm, *N* = 3, two male mice and one female mouse.

Since obvious corneal hyperplasia was observed in the H&E staining assay, immunohistochemistry was subsequently performed to characterize epithelial and stromal cell proliferation and differentiation in the cornea. KI67 is a general biomarker for cell proliferation, and Pax6 and Keratin12 are biomarkers for epithelial cell differentiation. The small number of KI67-positive cells in the cornea of control mice indicated that moderate epithelial cell proliferation and replacement occurred in the cornea under normal conditions. It is well-recognized that in normal corneas the limbal stem cells divide to produce daughter cells that proliferate, migrate, and differentiate to replace the lost cells [33]. However, a large number of KI67-positive cells were observed in the cornea of HE4-OE mice (Figure 4A), suggesting an increased epithelial cell proliferation. High levels of Pax6 (Figure 4B) and Keratin12 (Figure 4C) immunofluorescence were observed in the corneal epithelial cells of wild-type mice, indicating a normal differentiation. Interestingly, the corneal epithelial cells in HE4-OE mice displayed diminished expression of the two biomarkers, indicating possible loss of normal function by the regenerated epithelial cells. The low expression of the two biomarkers might be due to dedifferentiation or an unreplaced lose, e.g., by an increased apoptosis, of normal corneal epithelial cells. FSP1 (Fibroblast specific protein 1) is a commonly used biomarker for fibroblast activation [34]. The presence of many FSP1-positive cells near the base of epithelium as well as a few FSP1-positive fibroblasts in the stroma indicated fibroblast activation in the cornea of HE4-OE mice (Figure 4D).

### 3.5 Disorganized collagen fibers and decreased elastin fibers in the corneas of HE4-OE mice

**Figure 5:**
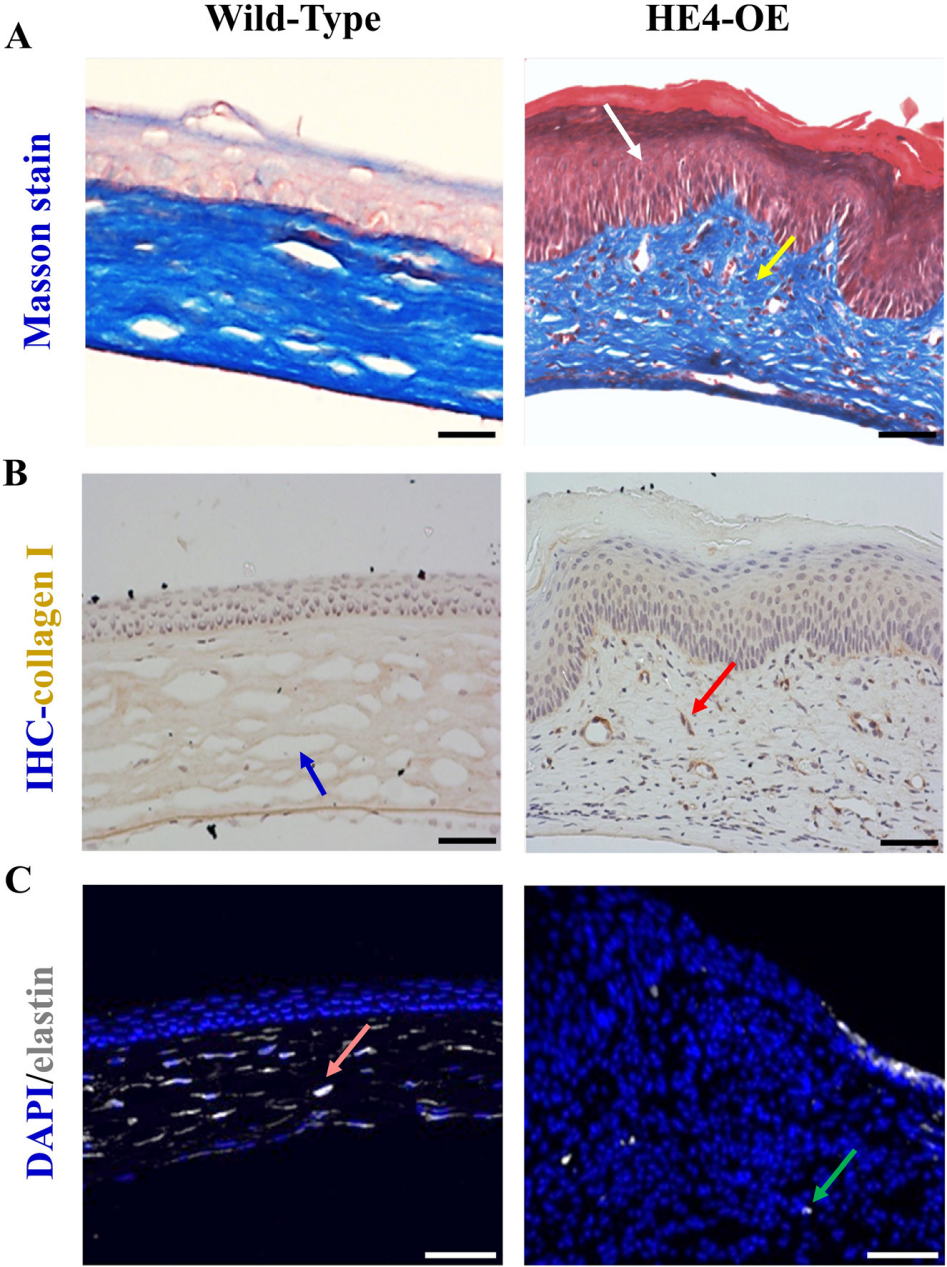
Alterations of the corneal collagen and elastin fibers in HE4-OE mice. The fiber structures in the corneal stroma were determined with Masson’s trichrome staining (A) and immunohistochemistry with specific antibodies against Collegan1 (B) or elastin (C). A. Results of Masson’s trichrome staining. Note the disorganized collagen fibers (yellow arrow) and the dramatic emergence of the dense red color staining (white arrow) in the corneal epithelium of 6-month-old HE4-OE mice. Scale bars = 50 µm, *N* = 3, two female mice and one male mouse. B. The collagen fibers in the corneal stroma (blue arrow) were well-organized and evenly distributed. The collagen fibers in the corneal stroma of 6-month-old HE4-OE mice became disorganized and unevenly distributed, often in a concentrated and speckled pattern (red arrow). Scale bars = 50 µm, *N* = 3, two female mice and one male mouse. C. The corneal elastin fibers were evenly distributed in 6-month-old wild-type mice (pink arrow). Only a few scattered corneal elastin fibers in a speckled form (green arrow) were present in HE4-OE mice. Scale bars = 50 µm, *N* = 3, two female mice and one male mouse. Note the much-increased cellular cells in the cornea of HE4-OE mice.

To determine the changes in the fibril components of the opaque corneal tissues from HE4-OE mice, Masson’s trichrome staining was performed. The collagen fibers were clearly visualized in blue color in the corneas of both wild-type and HE4-OE mice (Figure 5A). However, in the cornea of HE4-OE mice, collagen fibers became disorganized and arranged in chaotic orientations, which may contribute to the decreased transparency and opacification of the cornea. In addition, there was a dramatic change of the Masson staining pattern in the corneal epithelium. While the normal corneal epithelium displayed a light pink color, the tear film layer and entire epithelium of the cornea of HE4-OE mice with keratopathy exhibited a dark red color. Although in Masson’s trichrome staining the red color usually represents myofibers, the composition stained in red color in this case remained to be investigated.

Immunohistochemistry with anti-collagen1 antibody showed that while normal cornea possessed well-organized and evenly distributed collagen fibers, the collagen fibers in the cornea of HE4-OE mice were disorganized, and mostly in a concentrated, disrupted, and speckled pattern (Figure 5B). Moreover, elastin fibers were significantly reduced in the cornea of HE4-OE mice in comparison with that of wild-type mice (Figure 5C). In the corneas of HE4-OE mice, the few elastin fibers were in a scattered and speckled form, with some of them dislocated to the surface of epithelium. There were also much-increased cellular components in the corneal sections of HE4-OE mice. The increased cellular components and disordered collagen1/elastin fibers may affect the transparency as well as the function of the cornea in HE4-OE mice.

### 3.6 Emergence of vessel-like structures in the corneas of HE4-OE mice

**Figure 6:**
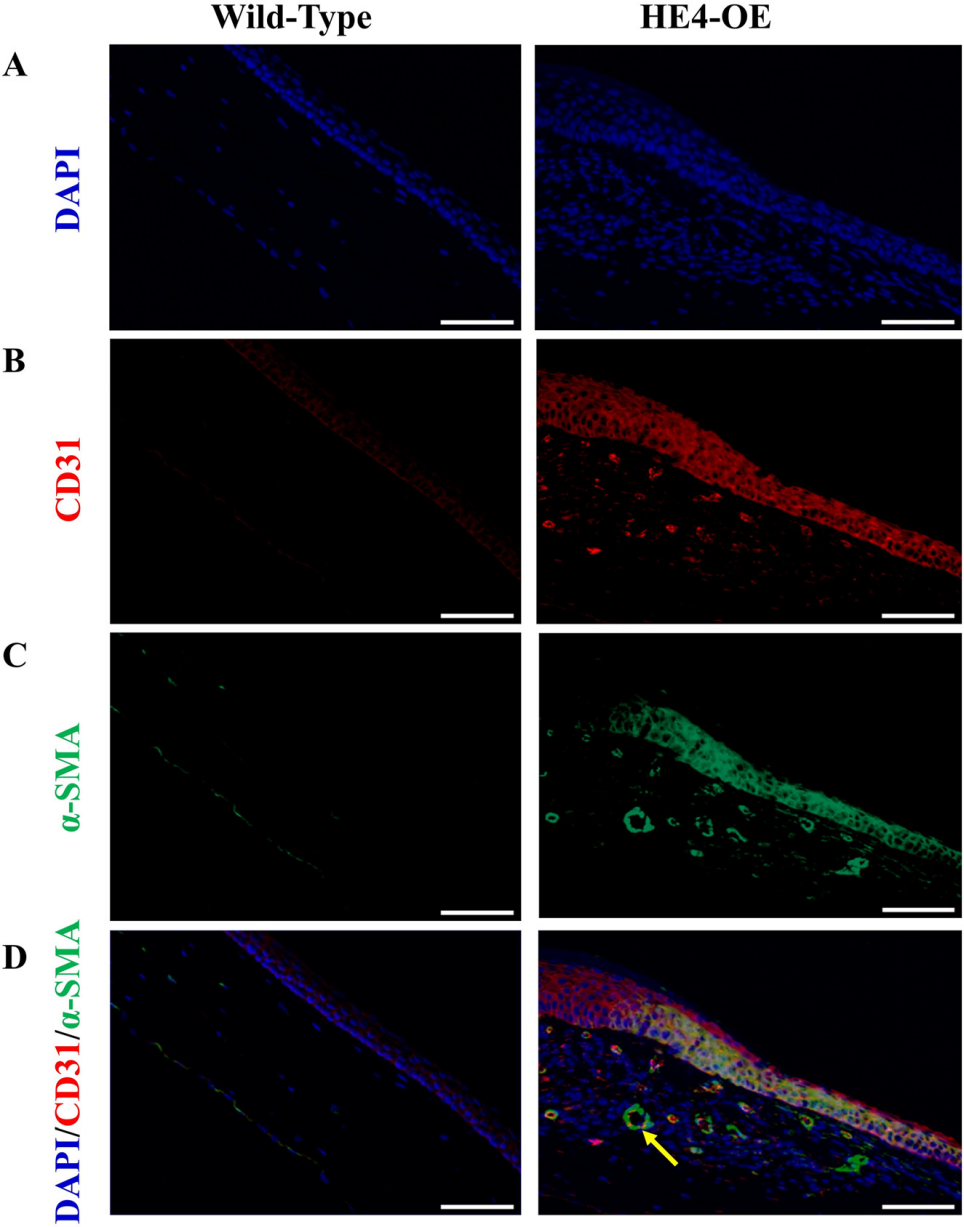
Emergence of vessel-like structures in corneas of HE4-OE mice. DAPI staining (A), and immunostaining for CD31 (B) and *α*-SMA (C) were performed in the same tissue section of the cornea, from 6-month-old wild-type (left column) and HE4-OE (right column) mice, respectively. D. Merged picture. The CD31 and *α*-SMA immunofluorescence signals are highly overlapped. Note the cellularly organized CD31- and *α*-SMA-positive cells, which formed vessel-like structures, in the corneal stroma of HE4-OE mice (yellow arrows). The CD31- and *α*-SMA-positive staining of the corneal epithelium in HE4-OE mice is indicated by white arrow. Scale bars = 50 µm, *N* = 3, two female mice and one male mouse.

The normal corneal tissue has no blood vessel to ensure corneal transparency, and the nourishment of the cornea depends on the aqueous humor. CD31, a vascular endothelial cell marker, and *α*-SMA (alpha-smooth muscle actin), a vascular smooth muscle marker, were employed to determine the angiogenic activity. As expected, an absence of neither CD31 nor *α*-SMA immunostaining signal was observed in the cornea of wild-type mice. However, obvious CD31 (Figure 6B) and *α*-SMA (Figure 6C) immunostaining was observed in the cornea of HE4-OE mice. The merged image displayed a high degree of colocalization of the CD31- and *α*-SMA-positivity as well as many vessel-like structures (Figure 6D), suggesting possible angiogenic activity in the corneal stroma of HE4-OE mice. Angiogenic activity in the cornea may contribute to the decreased cornea transparency and vision loss in HE4-OE mice. Curiously, strong CD31 as well as *α*-SMA immunofluorescence signals were observed in the corneal epithelium of HE4-OE mice, but not in the corneal epithelium of wild-type mice. The rise of CD31 and *α*-SMA positivity in the epithelium could be related to the regeneration/wound healing/scar formation process. Positive staining of the epidermal epithelial cells was previously observed in the mouse skin wound model [35].

### 3.7 Upregulation of HE4 expression in the corneal epithelium of the alkali burn model

**Figure 7:**
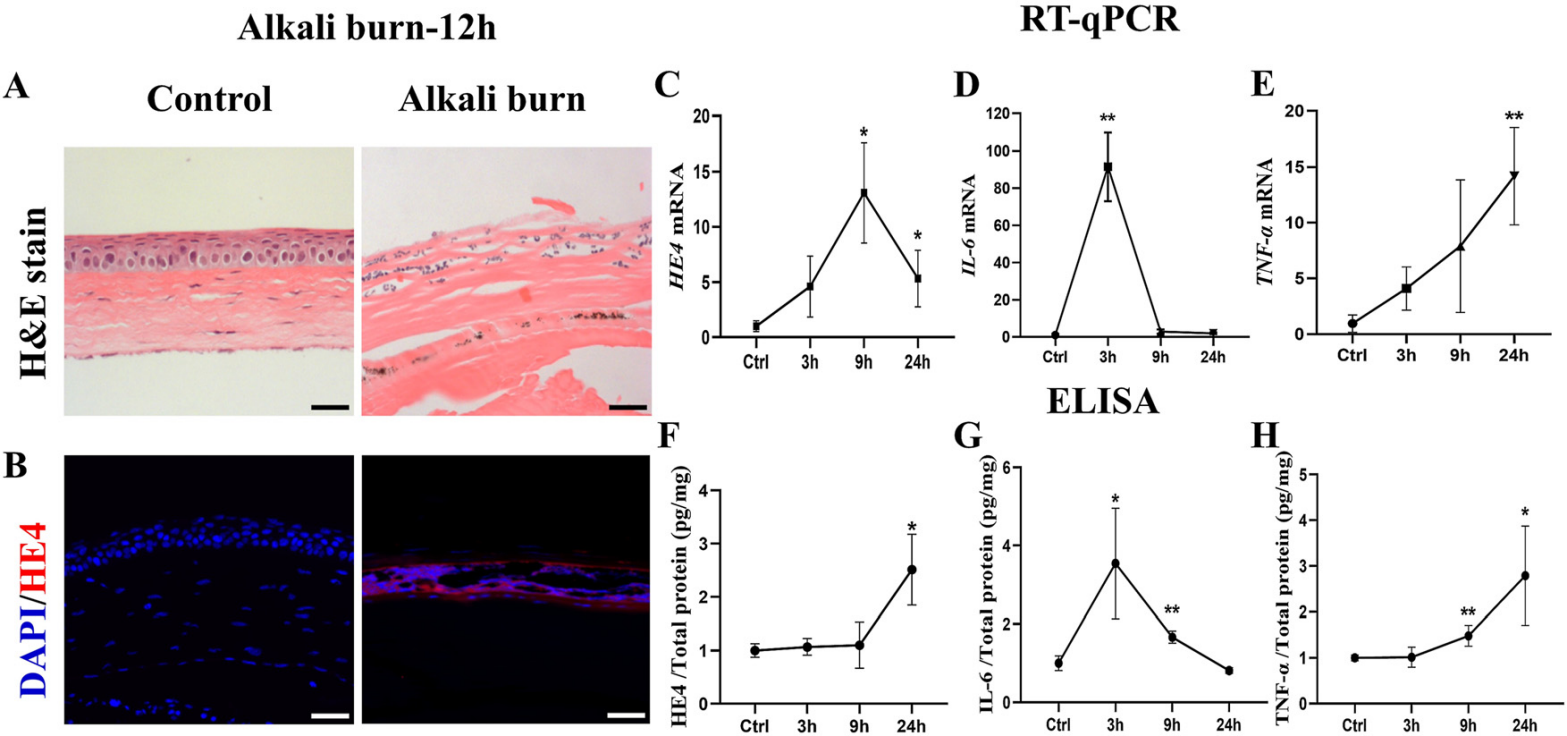
Dramatic upregulation of HE4 in the corneal epithelium following alkali burn. Alkali burn was performed in 12-week-old mice, and the control mice were treated with PBS. The corneal tissues were isolated at 12 h post-treatment for H&E staining and immunostaining. A. H&E staining showed that the corneal epithelium was disrupted and epithelial cells were dispersed. The stromal fibers were disorganized and disrupted. Scale bars = 50 µm, *N* = 3, two female mice and one male mouse. B. Results of immunohistochemistry with HE4 antibody, showing a dramatic increase of HE4 expression after alkali burn. Scale bars = 50 µm, sample size *N* = 3, two female mice and one male mouse, for each time point. Corneal tissues were harvested at 3 h, 9 h and 24 h after alkali burn. Real-time PCR was performed to determine mRNA (C, D and E), and ELISA was conducted to assess the protein levels of HE4, IL-6 and TNF-α (F, G and H). Alkali burn led to increased *HE4* mRNA and protein levels. The *HE4* upregulation was accompanied by the increase of pro-inflammatory factors *IL-6* and *TNF-α* mRNA as well as protein levels. *N* = 3, two female mice and one male mouse, for each time point. The data were expressed as mean ± SD. *p < 0.05, **p < 0.01.

The mouse corneal alkali burn model is a commonly used model for corneal injury, inflammation, and regeneration, and opacification [36], 37]. We applied this mouse model to investigate the correlation between HE4 expression and inflammation in the cornea. 12 h after burn, the cornea was isolated for histological analysis with H&E staining and immunostaining with HE4 antibody. The results of H&E staining showed that the protein fibers in the corneal stroma were broken and disorganized after alkali burn. The corneal epithelium was disrupted, and epithelial cells were dispersed (Figure 7A). Importantly, as shown in Figure 7B, the corneal damage led to a dramatic upregulation of HE4 expression in the epithelium. Quantitative analysis confirmed that the *HE4* mRNA level started to increase at 3 h after alkali treatment. The rise of *HE4* mRNA level reached a peak at 9 h before a downturn. The HE4 protein level was also increased at 9 h post-treatment (Figure 7F). The continued increase of HE4 protein level many hours after the 9 h point suggested post-translational modulation following alkali burn.

It is noteworthy that the upregulation of HE4 was accompanied by the elevation of pro-inflammatory factors of IL-6 and TNF-α, on both mRNA and protein levels (Figure 7D and E, G, H). The specific numerical values and *p*-values were listed in the supplementary information. These findings demonstrate a close relationship between corneal inflammation and upregulation of HE4. This observation, together with the keratitis phenotype of HE4-OE mice, strongly suggests that HE4 may play an important role(s) for the corneal inflammatory response.

## 4 Discussion

### 4.1 Inflammation is a constitutive part of the keratopathy in the HE4-OE model

Keratitis represents one of the pathological factors for corneal opacity [5]. Actually, all corneal opacity cases caused by ocular trauma, foreign bodies in the eye, and eye infection involve keratitis. Keratitis is indicated by increased levels of pro-inflammatory cytokines including interleukins and TNF-α in the cornea tissues or tears [38]. Elevated levels of IL-6, IL-1β and TNF-α are applied as defining features for keratitis animal models [39]. Systemic inflammatory conditions can also affect corneal structure and function. Ulcerative keratitis is the first sign of systemic necrotizing vasculitis in patients with Wegener’s granulomatosis [40], and a major ophthalmic manifestation of rheumatoid, an autoimmune disorder [41]. Keratitis of HE4-OE mice was supported by the observation of repeated eye-scratching behavior, redness of bulbar conjunctiva, and significant increase of corneal IL-6 and TNF-α levels. Many clinical cases of corneal opacity can be transient, and removal of foreign bodies in the eye or anti-microbial treatment can reverse the opacification process. The keratitis and corneal opacity phenotype of HE4-OE model appeared to be persistent and irreversible. Providing the role of HE4 in inflammation (discussed below) in non-corneal tissues, the ophthalmological phenotype of HE4-OE mice is most likely initiated by HE4 overexpression-triggered inflammation. Although no sign of infection was observed, secondary microbial infection could not be ruled out. It is noteworthy that besides keratitis, many HE4-OE mice had hair loss and the rough appearance of the skin especially at the area with hair loss, suggesting that these mice may suffer extensive immune/inflammatory anomalies in other tissues/organs as well.

### 4.2 The pro-inflammatory role(s) of HE4

It has been well-recognized that inflammatory conditions are associated with elevated circulatory HE4 levels. Indeed, when applying the HE4 serum test to cancer diagnosis, local and systemic inflammatory conditions should be taken into account as an interfering factor [42]. In non-malignant diseases, Zhang et al. observed that in TH2-high asthma, HE4 expression was markedly increased in the airway epithelial cells, plasma, and sputum [43]. Bene et al. demonstrated that the upregulation of HE4 in bronchial epithelial cells reflects a pro-inflammatory status of lung cystic fibrosis patients [44]. Zhan et al. reported that HE4 aggravates airway inflammation and remodeling in chronic obstructive pulmonary disease. Knockdown of HE4 expression in human bronchial epithelial cells alleviated whereas overexpression of HE4 facilitated, the cigarette smoke extracts-induced IL-6 expression, through affecting phosphorylation of NFκB-p65 [45]. In spite of circumstantial evidence in support of an HE4 role(s) in inflammation/immune function, the direct targets(s) of HE4 protein remain unidentified.

### 4.3 The pathogenesis of corneal opacity in human and in HE4-OE mice

Normal cornea does not contain blood vessel, and the corneal stroma contains well-organized and evenly distributed type-1 collagen fibers. Such structural arrangements ensure the transparency of the cornea [46]. But when the corneal epithelium is exposed to physical, chemical or biological hazards, epithelial cell proliferation, structural re-arrangement of collagen fibers, and angiogenesis occur during keratitis and wound healing, which affects the corneal function and normal vision [47]. We observed that the cornea of HE4-OE mice underwent extensive epithelial and stromal hyperplasia. The significantly increased KI67 and FSP1 expression and decreased Pax6 and Keratin12 expression indicated an enhanced proliferation of epithelial as well as stromal cells and abnormal epithelial cell differentiation. Moreover, the cornea of HE4-OE mice contained cellularly organized CD31-/α-SMA-positive cells, and the emergence of these vessel-like structures suggested possible presence of angiogenesis. Masson trichrome staining and collagen immunohistochemistry confirmed the accumulation and disorganization of collagen fibers. It was the combined effects by these changes that caused corneal opacity. In the alkali burn model of wild-type mice, the significant upregulation of HE4 on both mRNA and protein levels was associated with an immediate rise of IL-6 and TNF-α levels in the cornea. Overall, these findings have correlated HE4 overexpression to corneal inflammation. Although it is known that HE4 molecule possesses protease inhibitory activity, it is unclear if HE4 may exert a pro-inflammatory function via this biochemical activity. The current study is limited to the establishment and histological characterization of the keratitis and corneal opacity model, and the molecular mechanisms remain to be investigated.

### 4.4 Genetic mutations associated with corneal opacity

The HE4 overexpression-caused phenotype recapitulates the congenital corneal opacity, which occurs in 1 out of 5188 live births [48]. Kim et al. reported two female infants with an unremarkable perinatal history, but displayed photophobia, vascularizing keratitis and leucomatous corneal opacity in both eyes 3 months after birth [49]. Whole-exome sequencing identified a heterozygous c.1669C>T (p. Arg557Cys) pathogenic variant in the sterol regulatory element-binding protein 1 (SREBF1) gene. Corneal keratitis and opacity were observed in other mouse genetic models. Overexpression of protegrin 1 (PG1) in mice resulted in severe inflammation in the eye and corneal opacity [50]. Fucosyltransferases (Fut1) gene knockout mice displayed inflammatory responses in the ocular surface and Th1 cell activation in ocular draining lymph nodes (DLNs), and desiccating stress further aggravated the Th1 cell-mediated immune responses in DLNs and lacrimal gland, which led to severe corneal epithelial disruption and opacity [51]. Thus, keratitis and corneal opacity may represent a polygenic disorder, and the current studies of HE4-OE mice have enriched the spectrum of genes contributing to the disease. HE4’s endogenous protease inhibitory activity may be involved in keratopathy by participating in the regulation of tear proteases, corneal innate defense, and/or eye development. Interestingly, a spontaneous mutation in WFDC1 was found to be responsible for multiple ocular defects in cattle [29]. Specific expression of WFDC1 was detected in the lens, retina, and optic nerves of embryonic and adult mouse eyes, suggesting an essential role of WFDC1 in mammalian eye development.

### 4.5 Limitations of the current study

Since the HE4-OE mice suffer a low fecundity in both males and females [31], expansion of the strain has been difficult, and only three mice were included in each group; To be consistent, three mice were used in alkaline burn experiment as well. The phenotype studies on keratitis and tear film layer abnormality, opacity, and vision loss, were based on qualitative observation rather than quantitative measurement; In Masson’s trichrome staining (Figure 5), the identity and possible pathological role(s) of the component represented by the dense red color in the cornea of HE4-OE mice were undetermined. These limitations may affect the data quality.

## 5 Conclusions

It should be pointed out that while many HE4 studies have been focused on its applications to cancer management, and research efforts on HE4 functions are limited and scattered. The physiopathological role(s) of HE4 remains far from being fully understood. This study, for the first time demonstrates a specific role of HE4 in the ophthalmic physiology and disease. Overexpression of a single gene of HE4 is sufficient to cause corneal opacity. The findings raised a question concerning the possible pathological effects of the frequently fluctuating HE4 levels by many cancerous and non-cancerous conditions. Potentially, the HE4-OE model can be used to investigate the pathological mechanisms as well as treatment of keratitis and opacity.

## Supplementary Material

Supplementary Material
