# Supplementary material for: Overexpression of HE4/WFDC2 gene in mice leads to keratitis and corneal opacity
Source: Open Life Sci. 2025 Dec 29;20(1):20251234. doi: 10.1515/biol-2025-1234 (PMC13011896; doi:10.1515/biol-2025-1234)
Supplement: Supplementary file 1 — Supplementary Material [file j_biol-2025-1234_suppl_001.docx]

**Supplementary Material**

**Table S1. Primers for Real-Time PCR**

| Genes | Direction | Sequences | Size(bp) |
| --- | --- | --- | --- |
| GAPDH（mouse） | Forward | 5’-TGTGTCCGTCGTGGATCTGA-3’ | 77 |
|  | Reverse | 5’-CCTGCTTCACCACCTTCTTGA-3’ |  |
| WFDC2（mouse） | Forward | 5’-AACCAATTACGGACTGTGTGTT-3’ | 115 |
|  | Reverse | 5’-TCGCTCGGTCCATTAGGCT-3’ |  |
| IL-6（mouse） | Forward | 5’-CTCTGCAAGAGACTTCCATCCAGT-3’ | 72 |
|  | Reverse | 5’-GAAGTAGGGAAGGCCGTGG-3’ |  |
| TNF-α  (mouse) | Forward | 5’-AGGGTCTGGGCCATAGAACT-3’ | 103 |
|  | Reverse | 5’-CCACCACGCTCTTCTGTCTAC-3’ |  |

**Table S2. Numerical value for Figure 3 C**

|  | 1 | 2 | 3 | 4 | Mean | STDEV | *p* |
| --- | --- | --- | --- | --- | --- | --- | --- |
| wild-type | 0.819 | 0.666 | 1.220 | 1.295 | 1.000 | 0.265 |  |
| HE4-OE | 1.501 | 1.362 | 1.686 | 1.957 | 1.627 | 0.223 | 0.020 |

**Table S3. Numerical value for Figure 3 D**

|  | 1 | 2 | 3 | 4 | Mean | STDEV | *p* |
| --- | --- | --- | --- | --- | --- | --- | --- |
| wild-type | 1.091 | 0.953 | 0.891 | 1.065 | 1.000 | 0.082 |  |
| HE4-OE | 2.307 | 1.891 | 1.320 | 1.150 | 1.667 | 0.460 | 0.048 |

**Table S4. Numerical value for Figure 7 C**

|  | 1 | 2 | 3 | Mean | STDEV | *p* |
| --- | --- | --- | --- | --- | --- | --- |
| Ctrl | 0.851 | 1.540 | 0.608 | 1.000 | 0.395 |  |
| 3h | 3.838 | 2.297 | 6.655 | 4.263 | 1.804 | 0.067 |
| 9h | 11.752 | 18.121 | 9.349 | 13.074 | 3.701 | 0.010 |
| 24h | 8.095 | 4.814 | 3.057 | 5.322 | 2.088 | 0.045 |

**Table S5. Numerical value for Figure 7 D**

|  | 1 | 2 | 3 | Mean | STDEV | *p* |
| --- | --- | --- | --- | --- | --- | --- |
| Ctrl | 0.638 | 0.341 | 2.021 | 1.000 | 0.732 |  |
| 3h | 97.719 | 70.734 | 106.039 | 91.497 | 15.070 | 0.001 |
| 9h | 1.564 | 3.414 | 3.596 | 2.858 | 0.918 | 0.089 |
| 24h | 2.376 | 2.010 | 1.416 | 1.934 | 0.395 | 0.188 |

**Table S6. Numerical value for Figure 7 E**

|  | 1 | 2 | 3 | Mean | STDEV | *p* |
| --- | --- | --- | --- | --- | --- | --- |
| Ctrl | 0.314 | 0.727 | 1.818 | 1.000 | 0.666 |  |
| 3h | 5.460 | 4.968 | 1.876 | 4.303 | 1.665 | 0.060 |
| 9h | 14.742 | 3.845 | 5.123 | 8.293 | 5.104 | 0.116 |
| 24h | 15.109 | 9.427 | 18.017 | 14.883 | 3.743 | 0.007 |

**Table S7. Numerical value for Figure 7 F**

|  | 1 | 2 | 3 | Mean | STDEV | *p* |
| --- | --- | --- | --- | --- | --- | --- |
| Ctrl | 1.092 | 0.857 | 1.051 | 1.000 | 0.102 |  |
| 3h | 0.980 | 1.247 | 0.974 | 1.067 | 0.127 | 0.593 |
| 9h | 0.603 | 1.377 | 1.320 | 1.100 | 0.352 | 0.720 |
| 24h | 2.924 | 2.866 | 1.747 | 2.512 | 0.542 | 0.018 |

**Table S8. Numerical value for Figure 7 G**

|  | 1 | 2 | 3 | Mean | STDEV | *p* |
| --- | --- | --- | --- | --- | --- | --- |
| Ctrl | 1.206 | 0.844 | 0.950 | 1.000 | 0.152 |  |
| 3h | 1.953 | 4.032 | 4.644 | 3.543 | 1.152 | 0.036 |
| 9h | 1.629 | 1.534 | 1.831 | 1.665 | 0.124 | 0.009 |
| 24h | 0.897 | 0.751 | 0.796 | 0.815 | 0.061 | 0.184 |

**Table S9. Numerical value for Figure 7 H**

|  | 1 | 2 | 3 | Mean | STDEV | *p* |
| --- | --- | --- | --- | --- | --- | --- |
| Ctrl | 1.028 | 1.059 | 0.913 | 1.000 | 0.063 |  |
| 3h | 0.780 | 1.083 | 1.206 | 1.023 | 0.179 | 0.871 |
| 9h | 1.594 | 1.719 | 1.192 | 1.502 | 0.225 | 0.038 |
| 24h | 2.101 | 4.064 | 2.260 | 2.808 | 0.890 | 0.046 |
